# Effect of Weaving Structures on the Water Wicking–Evaporating Behavior of Woven Fabrics

**DOI:** 10.3390/polym12020422

**Published:** 2020-02-12

**Authors:** Min Lei, Yuling Li, Yanping Liu, Yanxue Ma, Longdi Cheng, Yue Hu

**Affiliations:** College of Textiles, Donghua University, Shanghai 201620, China; 1185046@mail.dhu.edu.cn (M.L.); liuyp@dhu.edu.cn (Y.L.); yxma@dhu.edu.cn (Y.M.); ldch@dhu.edu.cn (L.C.); 18317157136@163.com (Y.H.)

**Keywords:** water transfer, wicking, evaporating, weave, woven fabric

## Abstract

Water transfer through porous textiles consists of two sequential processes: synchronous wicking–evaporating and evaporating alone. In this work we set out to identify the main structural parameters affecting the water transfer process of cotton fabrics. Eight woven fabrics with different floats were produced. The fabrics were evaluated on a specially designed instrument capable of measuring the water loss through a vertical wicking process. Each test took 120 min, and two phases were defined: Phase I for the first 10 min and Phase II for the last 110 min according to wicking behavior transition. Principal components and multivariate statistical methods were utilized to analyze the data collected. The results showed that Phase I dominated the whole wicking–evaporating process, and the moisture transfer speed in this phase varied with fabric structure, whereas the moisture transfer speeds in Phase II were similar and constant regardless of fabric structure. In addition, fabric with more floats has high water transfer speed in Phase I due to its loosened structure with more macropores.

## 1. Introduction

As one of the most important properties relating to textile materials, thermal comfort affects human thermo-physiological responses and depends heavily on heat and moisture transfer capability [1]. To provide a cooler and drier feeling in hot and wet environmental conditions, water transfer is crucial for human beings, especially for athletes, workers, and firefighters [2,3]. There are two sequential processes during water transfer through porous textiles: synchronous wicking–evaporating and evaporating alone. This complex process is affected by the fiber hydrophilicity/hydrophobicity, fabric structure, and finishing [4]. In the case of excessive perspiration, clothes are saturated with air and moisture. The relatively slow action of wicking and evaporation could make people feel stickiness and discomfort, which consequently influences the fatigue level of the wearer and increases skin surface temperature [5]. Therefore, evaluation of the water transport ability of textiles is important for the optimization of sportswear, functional clothing, or other healthcare products.

**Figure 1:**
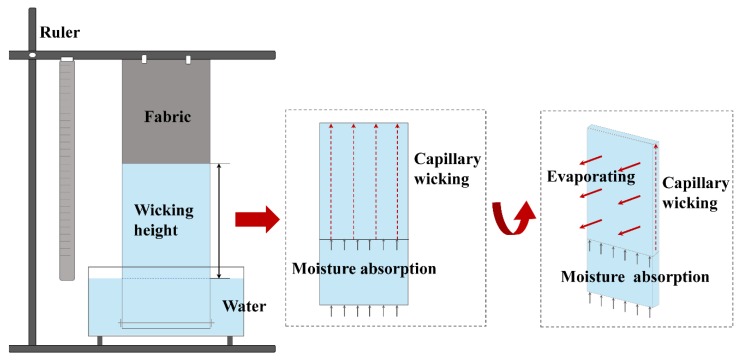
Water wicking and evaporating occur simultaneously in a typical vertical wicking test.

As a widely used wicking measurement method, the strip vertical wicking test was first proposed by Ghali [6]. Previous studies focused mostly on physical models [7,8] and measurements [9,10] of wicking behavior in yarn and textiles and took the maximum wicking height/distance as a key moisture transfer index [11,12,13,14,15,16,17,18]. It was previously observed that wicking and evaporation occur simultaneously when a fabric is partly immersed in water [19] as illustrated in Figure 1. Water transfer begins spontaneously, driven by capillary force, and evaporation takes place at the same time when the fabric is in contact with water [20]. Previous studies concerned with wicking–evaporating have been carried out on nontextile materials [19,20,21,22]. A twill metallic weave was studied and an evaporation rate model for predicting wicking height was proposed by Fries using Lucas and Washburn equations, indicating that the effect of evaporation in the wicking process is evident [21]. The sample with high evaporating rate had low total wicking height when compared with the sample with low evaporation. In this regard, the widely used strip vertical wicking test method cannot determine the quantity of water transferred by the fabrics, which is composed of wicking mass and evaporation mass. Evaporation and wicking are a combined issue, especially for the design of sportwear. It is crucial that the moisture be transferred away from the skin to the out-layer of garments where it should be evaporated quickly [23]. However, investigation considering both the wicking and evaporating performance of fabrics is still lacking. An in-depth understanding of how water is transferred in fabrics by wicking and evaporating, as well as identifying the factors affecting this wicking–evaporating process, is essential.

**Figure 2:**
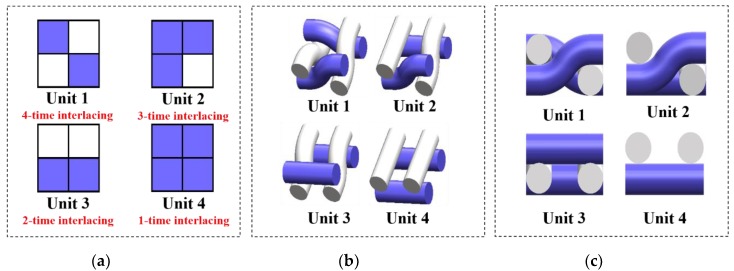
(**a**) Weave diagram, (**b**) 3-D structure, and (**c**) 3-D sectional structure of four basic units in woven fabrics.

Water transfer behavior is highly related to the geometry of fabrics [24], which depends on the density, weaves, thickness, and porosity [11,14,15,25]. The porosity of fabrics, determined by the structural weave [26,27], could induce changes in the structure, shape, and the number of capillary channels through the inter-fiber and inter-yarn pores [7,8,28], which affects the moisture transfer at various levels of capillary force [4,24]. It was found that the float and interlacing point, which are the two elements in a weave, can increase the whole water transfer to a certain degree [13,29]. Babu [13] discussed the effect of woven fabric weave factor on vertical wicking behavior and revealed that the rate of wicking increased with an increase of float, and the fabrics with evenly distributed floats showed slow wicking rate with horizontally striped weaves. A horizontal wicking test was conducted on woven fabrics with a designed sweat transfer tester [29], which suggested that fabrics with more floats had higher water transfer rate because floats contain more air space than plain weave. This finding results from the different topographical structure induced by differences in the weave of the yarn interlacing of fabrics, which is responsible for capillary actions and the water transfer capacity of woven fabrics [30]. Common weaves with varied topographical characteristics were used to conduct water transfer tests [31]. It was concluded that the topography of the fabrics controls the transfer rate of water drops in the meso length scale. The plain weave possessed the lowest transfer speed when compared with twill and panama [31]. However, studies on the water transfer behavior of woven structures taking the percentage influence of different weaving elements into consideration are rare, and the fabric structures employed in these are basic weaves including plain, twill, satin, etc. [13,16,29,30,31,32,33]. As shown in Figure 2, depending on the weave (the order of interlacement), four types of basic units are possible in one-layer woven fabrics, and the float lengths of them are different [34,35]. Studies revealing evaporation behavior in the wicking process in textiles, which is of great importance to understanding water transfer in textiles, are still lacking.

In this study we aimed to reveal the water transfer process on woven fabric and to identify its main influencing structural parameters. We firstly developed a novel and simplified gravimetric measurement to detect the weight loss of mass transfer during the fabric wicking process. Two phases of mass transfer were adopted to evaluate the weight loss speed. Besides this, the relationships between structural parameters and wicking–evaporation behavior were further studied. It is expected that the findings from this study could enhance our understanding about the effect of weave design on the wicking–evaporating behavior of woven fabrics.

## 2. Materials and Methods

### 2.1. Samples and Materials

All weave types are composed of interlacing points and non-interlacing points. As presented in Figure 2, for a unit of 2 × 2 weave type, there are four types of interlacing methods, forming four units. Adjusting the proportions of the four units in a weave can create different structures with varied floats. For example, a weave formed with Unit 1 has no float, but a weave with Unit 1 and Unit 2 has more floats. Since the weave pattern influences the wicking and evaporating behavior of fabrics, the effects of the four weave units and their arrangements on moisture transfer properties were investigated. Eight fabrics were designed and produced with different proportions of weave units, and the related weave parameters are listed in Table 1. U1, U2, U3, and U4 are the proportions of Unit 1 to Unit 4, respectively.

Two parameters were defined here to account for the number of floats in a weave: the crossing-over firmness factor (CFF) and the floating yarn factor (FYF).

The crossing-over firmness factor is defined by [36]

(1) $$\mathrm{CFF}=\frac{\text{Number of crossrepeating }-\text{over lines in the complete }}{\text{Number of interlacing points in the complete repeat }}$$

The crossing-over line number is the number of interlacing times in a complete repeat; for example, when the warp yarn changes from over to beneath the weft yarn, or vice versa in the warp direction. In the case of the plain weave, there are eight crossing-over lines in a complete repeat and four interlacing points. Therefore, the CFF becomes 2.0.

The floating yarn factor [36] is expressed as

(2) $$\mathrm{FYF}=\frac{(\mathrm{FL}-1)\times \text{existing number of float in the complete repeat }}{\text{number of interlacing points in the complete repeat }}$$

where FL is the floating length.

**Figure 3:**
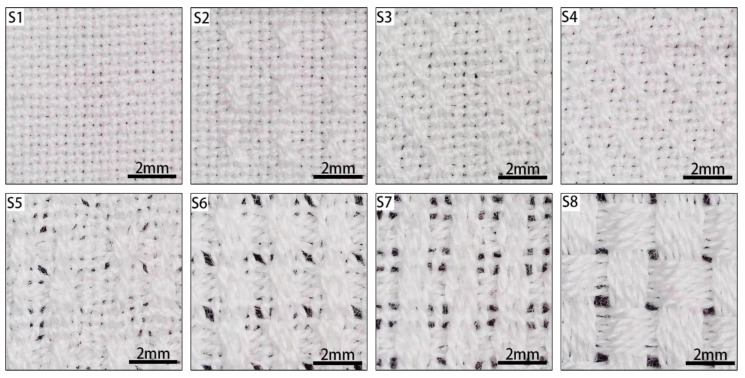
Images of woven fabrics with different proportions of weave units.

All samples were made of 100% cotton 20/2 (Ne) count yarns on a TNY501C-20 automatic rapier loom. The warp and weft densities were 24 picks/cm for all samples during the weaving process. Subsequently, the samples were washed in NaOH solution (0.8 wt %, 1:50) at 95–100 °C for 60 min. Then, the samples were washed twice with distilled water to remove dust or grease left on the surface or in the interstices. Images of the woven fabrics with different proportions of weave units are shown in Figure 3.

### 2.2. Testing Methods

The physical and structural properties of the eight woven fabrics were characterized. The fabric areal density (mass per unit area) and thickness were measured according to EN ISO 5084-2002 and ISO 3801-1977, respectively. Images of the yarn diameter and morphology were captured with a digital microscope at 5× magnification. The warp and weft yarn diameters were 0.227 mm and 0.245 mm, respectively (the density of the cotton fiber was 1.52 g/m^3^). In addition, the firmness factor, bulk porosity, and average pore hydraulic head radius of the woven fabrics were calculated. The surface porosity was measured through image analysis. All the measured and calculated results are listed in Table 2.

**Figure 4:**
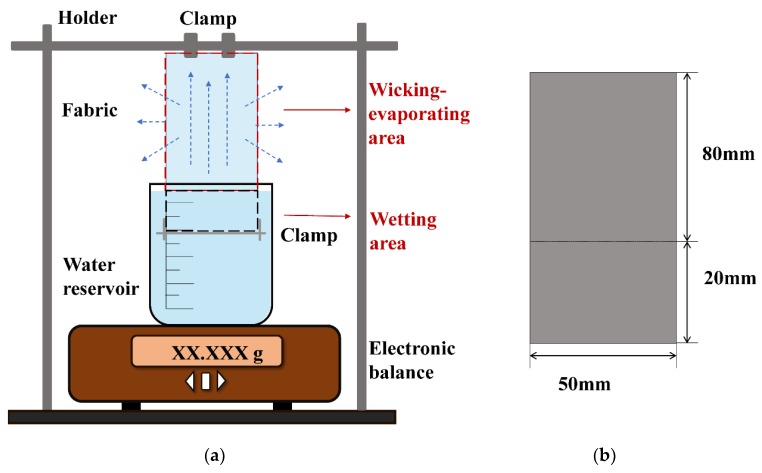
(**a**) Schematic diagram of the setup. (**b**) Horizontal and vertical size of the test sample.

To assess the wicking–evaporating behavior, a setup capable of weight loss detection was built based on vertical wicking valuation. A schematic diagram of the setup is shown in Figure 4. The setup consisted of the following:

● An electronic balance with an accuracy of 0.001 mg to record the wicking–evaporating-induced weight loss versus time;
● A liquid reservoir with a capacity of 250 mL distilled water to supply water to the fabric;
● An iron holder to mount the test samples; and
● A clamp to add pre-tension to the lower end of the sample and to keep samples free of wrinkles.

The setup was placed in standard atmospheric conditions (temperature 20 ± 2 °C, relative humidity 65% ± 5%). All the samples were conditioned in the same atmospheric conditions for 48 h prior to testing.

The test procedure was as follows: We mounted the sample onto the iron support and immediately immersed it into the liquid reservoir with 20 mm immersion. The measurements were repeated three times, and weight loss was recorded using the electronic balance every one minute in Phase I and every ten minutes in Phase II. During the test, the sample was in an environment which was composed of air and evaporation vapor of the test liquid.

## 3. Results and Discussion

### 3.1. The Wicking–Evaporating Behavior

**Figure 5:**
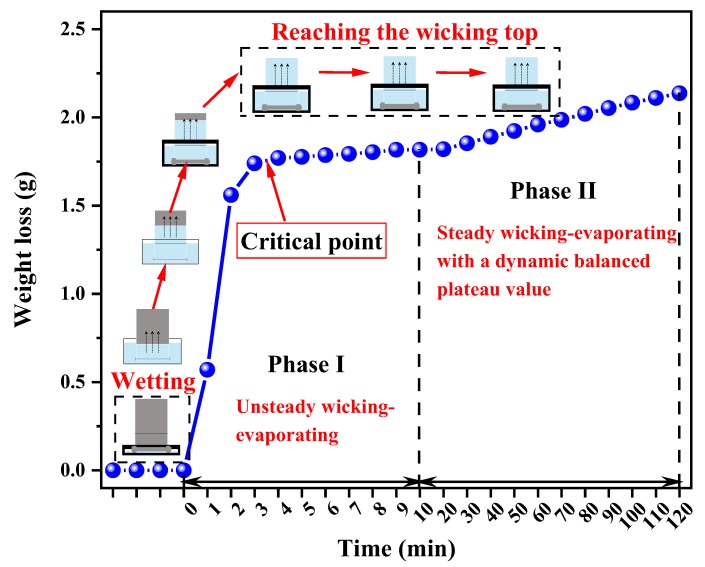
Typical curve of the wicking–evaporating process in woven fabrics within two phases, taking Sample 8 as an example.

Figure 5 illustrates the wicking–evaporating process. The curve is divided into two parts, describing the fabric from imbibition to evaporation: Phase I, unsteady wicking and evaporation in the initial 10 min with water transfer speed *S*_1_; Phase II, steady wicking–evaporating from 10 to 120 min with water transfer speed *S*_2_. The whole observed process is in good accordance with that in previous research [7,21].

Firstly, the fabrics were brought into contact with the water, inducing the wetting process. Then, capillary suction took place at the liquid–gas interface at the surface as well as within the inner of the porous medium because of spontaneous liquid transfer via wicking [20]. Evaporation occurred simultaneously when water started to wick into the fabrics [21]. Due to the vapor pressure of the test liquids, evaporation occurs within a layer of vapor over the weave surface. While the liquid rose in the porous woven fabric, water wicked inside the fabric was exposed to the ambient atmosphere at the fabric surface pores. If the surrounding gas is not saturated with the liquid vapor, evaporation out of surface pores can occur [19]. Due to the equilibrium state between capillary and hydrostatic pressure in the presence of evaporation, the wicking front reached the top of the sample at 80 mm, and the rate of water transfer remained constant. This situation was maintained until the test finished.

### 3.2. The Effect of Wicking–Evaporating Behavior

**Figure 6:**
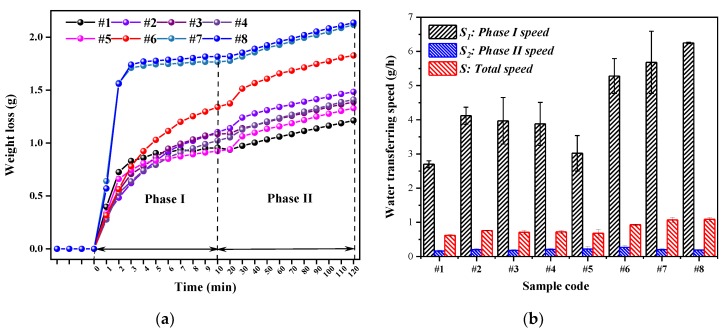
Measurement of wicking–evaporating: (**a**) weight loss versus time and (**b**) water transfer speeds for the eight samples.

The moisture transfer speeds of the eight samples were significantly different in Phase I (through analysis of variance, SIG = 1.05 × 10^−6^). It can be observed from Figure 6a that their overall behavior followed the same tendencies: each of the eight samples reached its critical point during Phase I, and the water transfer speed finally had a dynamic balanced plateau value after achieving the longest wicking distance. In Phase II, the ANOVA (Analysis of Variance) indicated that the weave pattern has no significant influence (SIG = 0.108) on transfer speed. Figure 6b shows that the *S_1_* and *S* (total water transferring speed during 120 min) values shared a similar trend and varied significantly among the eight samples, whereas *S*_2_ differed slightly from 0.16 to 0.27 g/h. Due to the large contribution of wicking to water transfer in Phase I, the total water transfer speeds were significantly different among the samples, with SIG = 1.72 × 10^−6^, which suggests that wicking dominates the whole water transfer process in the vertical wicking–evaporating test.

According to the different characters of fabric water transfer, the fabrics can be divided into two groups. Group 1 contains S1–S5, whose floats are distributed evenly throughout the entire fabric surface. Their total water transfer speeds varied slightly from 0.62 to 0.72 g/h. Group 2 includes vertically striped fabrics S6–S8, whose total water transfer speeds (0.93–1.09 g/h) were higher than those of Group 1. The floats of the warps are vertically striped and distributed throughout the entire fabric surface, and this relatively nonuniform structure of float combined with interlacing may induce different sizes of macropores inside and on the surface of fabrics, which can be explained by pore network model theory [37]. When the pore diameters in a porous medium differ more from one another (from macropore to micropore), the unevenness degree of the pore size distribution is greater and the balance of the pore network changes, inducing a relatively high capillary force [38]. The liquid moves into a capillary tube when it is subject to capillary pressure, like the differential pressure across the liquid–air interface, because of the curvature of meniscus in the narrow confines of the capillary [39]. For instance, the involvement of Unit 1 will destroy the connectivity points, resulting in low capillary force in the vertical direction. Consequently, the pore network in fabric S1 formed with 100% Unit 1 is uniform and smooth with low capillary pressure, having the slowest speed of total water transfer at 0.62 g/h. By contrast, the structure of macropores and connective capillary tubes created with the long floats and few interlacings of Unit 4 can directly induce high-speed water transfer phenomena due to its relatively nonuniform capillary channels. Hence, the water transfer speed of S8 (which is composed of 69.44% Unit 4) in Phase I was the highest among all samples. Besides this, fabrics with high porosity transfer moisture more quickly than do those with relatively low porosity before the equilibrium state, which occurs at the macropore scale. The results in our study showed good accordance with those of a study on the capillaries in different macropore textile structures [40].

At the equilibrium state in Phase II, the wicking front reaches its extreme distance at the top of the sample, which leads the fabrics to be fully covered with water. The water transfer speed showed weakly significant differences between the eight samples during Phase II. The fabric surface, behaving as a fully wetted surface, was sufficiently and finely divided when at the wicking top [19]. In this condition, the water transfer rate from a fully wet surface is basically identical to that of a free liquid surface. This is the screening phenomenon [41] of saturated porous media, which can help to explain why the water transfer rate of a partially solid and partially liquid surface (samples in Phase I condition) is different from that of the same surface fully saturated with water (samples in Phase II condition).

Pearson’s correlation analysis was used to assess the strength of the association between total water transfer speed and the investigated variables, and the results are listed in Table 3. FF (firmness factor), AD (areal density), and TC (thickness) are basic structural parameters; BP (bulk porosity), SP (surface porosity), and HH (hydraulic head diameter) are pore parameters. A positive value in Table 3 indicates that the variables in the respective pair increase or decrease simultaneously, whereas a negative value indicates that as one variable increases, the other decreases, and vice versa. The strength of association between the variables increases as the absolute value increases. A value of 1 means that the two variables are completely associated. Among all the variables, the FYF had the largest correlation (r = 0.897 *), followed by CFF (r = 0.869 **) and proportion of Unit 1 (r = 0.869 **), which means that total water transfer speed has a strong association with the weave. On the one hand, it is certain that the weave parameters themselves have a large correlation value. This is exemplified by the observation that when the proportion of Unit 1 decreases, the CFF declines. If the proportion of Unit 4 increases, which is representative of the longest floating length among the four basic units, the calculated value FYF will rise. On the other hand, it should be noted that bulk porosity had the highest absolute correlation coefficients: 0.912, 0.945, and 0.929 with CFF, FYF, and U1, respectively. It is considered that bulk porosity depends markedly on the weave parameters. Increase of the length floating point proportion by varying weave parameters creates a loosened structure with more macropores. Thus, air convection between the surroundings and the fabric inside can be enhanced, which helps air to take the free vapor out and accelerates evaporation.

### 3.3. Prediction of the Total Water Transfer Speed

To avoid disturbance by multicollinearity among variables, we performed a principal component analysis (PCA) on the data matrix and built a regression model on the principal components. PCA is a procedure of converting the observations of a set of possibly correlated variables into values of a set of linearly uncorrelated variables, called principal components, through an orthogonal transformation. The first two components, PC1 and PC2, out of six obtained PCs, were selected as new variables to build a regression model because they accounted for 94.34% of the relevant information, and those PCs with accumulated contribution rate of less than 0.9 were ignored in the analysis.

**Figure 7:**
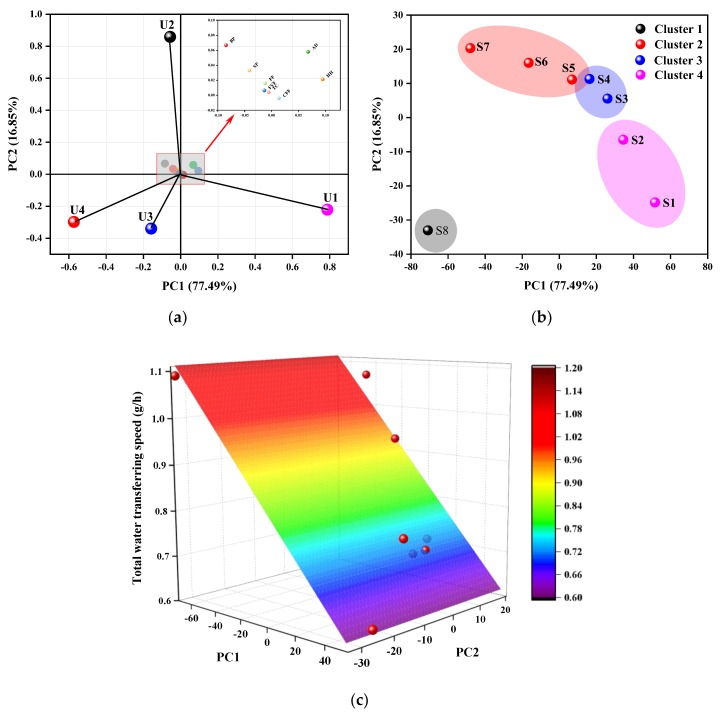
Position of the (**a**) 12 parameters, (**b**) samples clustered via the K-means method, and (**c**) scatter plot (red balls) and regression plane of the total moisture transfer speed in the PC1–PC2 coordinate system.

The principal component (PC), contribution, eigenvalue, contribution rate, and accumulated contribution rate after PCA processing are listed in Table 4. Every loading represents the correlation coefficients between the variables and the corresponding PC. The larger the absolute value of the coefficient, the better the variable can explain the corresponding PC. Figure 7a shows the correlations between the original variables and the first two PCs in the PC1–PC2 coordinate system. PC1 is mainly composed of the tightest U1 and loosest U4, which is highly relevant to the air convection and moisture transfer properties of fabric. U2 and U3 are the main components of PC2. It is evident that the proportions of the four units of weave explain most of the structural variation of the fabrics, which helps to prove that Unit 1 and Unit 4 are two main factors influencing the structure and performance of fabrics. The basic properties (AD, TC, FF), porosity parameters (HH, SP, BP), and the defined weave parameters (CFF, FYF) have a strong positive linear relationship as these three types of parameters are situated in proximity to one another. They contribute slightly to PC1 and PC2.

Figure 7b represents relationships within samples in the PC1–PC2 coordinate system, and the K-means clustering method was used to categorize the eight samples into four distinctive clusters with respect to the PC1 score. From left to right, the clusters generally show good consistency with the result from the proportion of Unit 1 versus samples. For instance, Cluster 1 has only one sample, S8, unlike all the other groups, and has the lowest proportion of Unit 1. S2 and plain weave S1 belong to the same cluster, Cluster 4, due to their possession of a relatively high number of interlacing points and fewer floats.

Based on the PCA method discussed above, a valid regression model can be established. In Figure 7c, the scatter plot and regression plane of the PCs versus total water transfer speed are plotted. The coefficient of determination of the binary linear regression of the first two PCs versus total water transfer speed was 0.8896. The ternary linear regression of PC1, PC2, and PC3 shows a high R^2^ value of 0.9158, which proves that the multiple linear regression model developed with the data observed in this study provides a good prediction of total water transfer speed. The equation to fit the relationship is given by

(3) S = 0.8208 − 0.004PC1 + 0.0052PC2 + 0.0028PC3

## 4. Conclusions

A wicking–evaporating test based on vertical wicking was used to investigate the water transfer behavior of cotton woven fabrics with different weaves. The following conclusions can be drawn from the analysis.

(1) There are two phases during the wicking–evaporating process: Phase I, with unsteady wicking and evaporating; and Phase II, with steady wicking–evaporating due to equilibrium between the capillary and gravity forces. Because of the uneven degree of capillary channels induced by different structures, the water transfer speed in Phase I varied with samples. However, it was constant regardless of weave structure in Phase II. Because of the screening phenomenon induced by continuous saturated conditions, the water transfer rate from a fully wet fabric surface is basically identical to that from a free liquid surface.
(2) Woven fabric with more float has more macropores, possessing nonuniform capillary channels and more air space for evaporating. Float can increase the total water transfer in the wicking–evaporating process more than interlacing points.

The findings of this study enhance the basic understanding of the effects of weave characteristics on the wicking–evaporating property of fabric. In future work, more weaves should be considered to investigate the effect of structural and weave parameters on water transfer. Theoretical analyses of coupled wicking–evaporation moisture transfer in fabrics are also needed.

## Figures and Tables

**Table 1:** Specifications of the eight woven fabric samples.

Sample Code	Weave	CFF	FYF	Proportion of Different Units (%)			
				U1	U2	U3	U4
S1	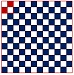	2.000	0.000	100.00	0.00	0.00	00.00
S2	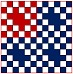	1.647	0.471	80.56	16.67	00.00	2.78
S3	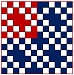	1.429	0.571	69.44	27.78	00.00	2.78
S4	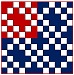	1.273	0.727	61.11	33.33	00.00	5.56
S5	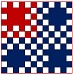	1.286	0.857	55.57	33.33	00.00	11.11
S6	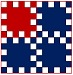	0.909	1.454	36.11	38.89	00.00	25.00
S7	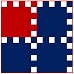	0.400	1.533	11.11	44.44	00.00	44.44
S8	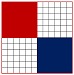	0.303	1.667	2.78	0.00	27.78	69.44

**Table 2:** Measured basic construction parameters of samples.

Sample Code	Areal Density (g/m^2^)	Thickness (mm)	Firmness Factor (%)	Bulk Porosity (%)	Surface Porosity (%)	Hydraulic Head (μm)
S1	168.73	0.55	84.56	0.8058	0.98	146.90
S2	170.50	0.68	83.26	0.8413	1.65	157.16
S3	166.75	0.67	81.95	0.8425	2.21	167.58
S4	172.58	0.69	84.11	0.8417	1.33	150.35
S5	176.67	1.01	86.26	0.8893	2.49	133.70
S6	171.58	1.04	85.45	0.8956	4.24	139.90
S7	160.42	1.08	85.30	0.9041	4.31	141.37
S8	163.58	1.25	84.38	0.9188	7.07	148.26

**Table 3:** Pearson’s correlation values among variables.

r	CFF	FYF	U1	U2	U3	U4	FF	AD	TC	BP	SP	HH	*S*
CFF	1	−0.972 **	0.980 **	−0.236	−0.586	−0.920 **	−0.325	0.552	−0.893 **	−0.912 **	−0.871 **	0.323	−0.869 **
FYF	-	1	−0.996 **	0.364	0.519	0.874 **	0.405	−0.409	0.917 **	0.945 **	0.856 **	−0.404	0.897 *
U1	-	-	1	−0.324	−0.539	−0.900 **	−0.378	0.485	−0.912 **	−0.933 **	−0.881 **	0.376	−0.869 **
U2	-	-	-	1	−0.559	−0.117	0.252	0.209	0.388	0.415	0.381	−0.248	0.514
U3	-	-	-	-	1	0.798 *	−0.009	−0.410	0.333	0.374	0.251	0.004	0.280
U4	-	-	-	-	-	1	0.302	−0.633	0.795 *	0.796 *	0.778 *	−0.302	0.708
FF	-	-	-	-	-	-	1	0.246	0.620	0.544	0.393	−1.000 **	0.520
AD	-	-	-	-	-	-	-	1	−0.357	−0.318	−0.650	−0.25	−0.301
TC	-	-	-	-	-	-	-	-	1	0.987 **	−0.916 **	−0.616	0.824 **
BP	-	-	-	-	-	-	-	-	-	1	0.885 **	−0.540	0.821 *
SP	-	-	-	-	-	-	-	-	-	-	1	−0.388	0.784 *
HH	-	-	-	-	-	-	-	-	-	-	-	1	−0.520

** significance at the 0.01 level. * significance at the 0.05 level.

**Table 4:** PCA results of amplitudes in the original parameter data.

**PC**	**Eigenvalue**	**Contribution Rate**	Accumulated Contribution Rate
**1**	1766.11	77.49	77.49
**2**	384.16	16.85	94.34
**3**	109.72	4.81	99.15
**4**	18.55	0.81	99.97
**5**	0.60	0.03	99.99
**6**	0.14	0.01	100.00
